# A Review of Orchard Canopy Perception Technologies for Variable-Rate Spraying

**DOI:** 10.3390/s25164898

**Published:** 2025-08-08

**Authors:** Yunfei Wang, Weidong Jia, Mingxiong Ou, Xuejun Wang, Xiang Dong

**Affiliations:** 1 School of Agricultural Engineering, Jiangsu University, Zhenjiang 212013, China; 2112416035@stmail.ujs.edu.cn (Y.W.); jiaweidong@ujs.edu.cn (W.J.); myomx@ujs.edu.cn (M.O.); 2 Key Laboratory of Plant Protection Engineering, Ministry of Agriculture and Rural Affairs, Jiangsu University, Zhenjiang 212013, China; 3 Chinese Academy of Agriculture Mechanization Sciences Group Co., Ltd., Beijing 100083, China; waxuju518@163.com

**Keywords:** orchard canopy, sensor technologies, variable-rate spraying, canopy perception, multimodal data fusion

## Abstract

With the advancement of precision agriculture, variable-rate spraying (VRS) technology has demonstrated significant potential in enhancing pesticide utilization efficiency and promoting environmental sustainability, particularly in orchard applications. As a critical medium for pesticide transport, the dynamic structural characteristics of orchard canopies exert a profound influence on spraying effectiveness. This review systematically summarizes recent progress in the dynamic perception and modeling of orchard canopies, with a particular focus on key sensing technologies such as LiDAR, Vision Sensor, multispectral/hyperspectral sensors, and point cloud processing techniques. Furthermore, it discusses the construction methodologies of static, quasi-dynamic, and fully dynamic canopy modeling frameworks. The integration of canopy sensing technologies into VRS systems is also analyzed, including their roles in spray path planning, nozzle control strategies, and precise droplet transport regulation. Finally, the review identifies key challenges—particularly the trade-offs between real-time performance, seasonal adaptability, and modeling accuracy—and outlines future research directions centered on multimodal perception, hybrid modeling approaches combining physics-based and data-driven methods, and intelligent control strategies.

## 1. Introduction

As global agriculture advances toward greater efficiency, sustainability, and intelligence, precision agriculture has become a key strategy for improving yields, optimizing inputs, and reducing environmental impact. In orchards, pest and disease control are essential for ensuring fruit quality and productivity. Spraying systems, the most widely used crop protection method, directly affect pesticide efficiency and ecological safety [1]. However, conventional uniform spraying relies on fixed parameters and overlooks the spatial heterogeneity of crop structures and varying pesticide needs, often leading to excessive chemical use, drift pollution, and inconsistent control effectiveness.

Variable-rate spraying (VRS) systems aim to overcome these limitations by dynamically adjusting spray volume, nozzle status, and spray trajectories based on the perceived structural, physiological, and density characteristics of crop targets [2,3,4]. As a result, VRS has become a key direction in the development of intelligent orchard spraying equipment. The core principle is “spray as needed,” whereby pesticide application parameters are adapted to the real-time protection requirements of the crops, achieving optimal efficacy with minimal input.

The effective implementation of VRS systems hinges on accurate perception and dynamic modeling of the crop canopy—the primary interface for pesticide–air interactions. Orchard canopies exhibit highly complex spatial structures, including layered distributions, interlaced branches and leaves, and significant density variations. Moreover, they respond dynamically to wind and undergo seasonal growth changes, highlighting their spatiotemporal variability [5,6,7,8]. These coupled “structure–dynamics–environment” characteristics play a decisive role in governing droplet transport pathways, penetration efficiency, and deposition patterns during spraying operations. Therefore, developing scalable, high-precision, and dynamic canopy perception and modeling techniques remains one of the most critical challenges in building intelligent VRS systems.

In recent years, significant progress has been made in orchard canopy perception, driven by rapid advancements in sensing and modeling technologies such as Light Detection and Ranging (LiDAR), visual imaging, spectral sensing, point cloud reconstruction, and deep learning [9,10,11,12]. LiDAR provides high-resolution 3D point cloud data that enables accurate reconstruction of tree volume, contour, and spatial architecture [13,14,15,16]. Multispectral and hyperspectral imaging can capture physiological states and the spatial distribution of pests and diseases within the canopy [17,18,19,20,21]. Visual imagery, when combined with semantic segmentation and object detection algorithms, allows for the precise identification of leaves and fruits [22,23,24,25,26,27,28]. Additionally, the integration of multi-sensor fusion techniques [29,30,31,32] with deep learning approaches [33,34,35] has significantly advanced the automation and intelligence of data-driven canopy modeling processes. Together, these technologies provide the foundation for intelligent decision-making in key components of variable-rate spraying (VRS) systems, including target identification, spray path planning, and nozzle control.

Despite these advancements, several challenges remain. On one hand, the spatiotemporal variability of orchard canopies limits the applicability of static models in dynamic field environments. Wind-induced collective vibration and structural deformation of leaf clusters can markedly alter droplet trajectories and deposition outcomes. On the other hand, the heterogeneity and unstructured nature of sensor data complicate data fusion and structural modeling. Moreover, existing models often struggle with issues related to real-time performance, generalizability, and seasonal adaptability. From an application perspective, another critical challenge lies in embedding perception outputs into VRS systems to enable closed-loop coupling between operational parameters and canopy structure.

To address these issues, this review aims to systematically examine the key technological pathways for dynamic perception and modeling of orchard canopies. The discussion is structured around three dominant modeling strategies—static, quasi-dynamic, and fully dynamic frameworks. We analyze their integration mechanisms, application modes, and technical bottlenecks within VRS systems, and further explore the interconnections among canopy structure, spray control, and droplet transport. Finally, we propose future research directions that emphasize multimodal data fusion, hybrid modeling approaches combining physics-based and data-driven methods, and unified frameworks for intelligent decision-making. This review is intended to provide theoretical insights and practical guidance for the development and deployment of intelligent orchard spraying systems.

## 2. Key Sensors for Canopy Perception

### 2.1. LiDAR (Light Detection and Ranging)

Light Detection and Ranging (LiDAR) is an active spatial sensing technology that emits laser pulses and measures the time it takes for the reflected signals to return, thereby calculating the distance to target objects and generating high-precision three-dimensional point cloud data [36,37,38]. LiDAR systems are characterized by their high spatial resolution, strong resistance to ambient light interference, and excellent adaptability to various environmental conditions. These advantages make LiDAR one of the most widely used sensor types for perceiving orchard canopy structures [39,40,41]. LiDAR enables the detailed reconstruction of canopy architecture, including branch distribution, leaf layering, and canopy volume. The core performance specifications of commonly used LiDAR systems in orchard applications are summarized in Table 1.

From the perspective of ranging mechanisms, LiDAR systems can be classified into three main types: pulsed time-of-flight (Pulsed ToF), amplitude-modulated continuous wave (AMCW), and frequency-modulated continuous wave (FMCW). Pulsed ToF LiDAR, the most widely used variant in agricultural applications, features a relatively simple architecture and a long measurement range, making it well-suited for mid- to long-distance modeling of orchard canopies. AMCW systems determine distance by modulating the intensity of the emitted light; they are more compact and energy-efficient, making them suitable for short- to medium-range structural sensing. FMCW LiDAR employs frequency modulation of the laser beam to achieve high-precision distance measurement and has the additional capability of simultaneously measuring velocity. However, FMCW systems are more complex and typically more expensive.

Based on imaging strategies, LiDAR systems can be classified into mechanically rotating, MEMS mirror-based, optical phased array (OPA), and flash solid-state types. Mechanically rotating LiDAR offers a wide field of view and stable data output, but its large size and limited operational lifespan constrain its applicability. MEMS-based LiDAR utilizes micro-electromechanical mirrors to steer the beam, featuring compact dimensions and low power consumption, making it well-suited for lightweight platforms. OPA systems steer laser beams electronically without moving parts, representing a promising pathway toward fully solid-state integration. Flash LiDAR captures entire scenes in a single frame with high imaging speed and no mechanical components, achieving efficient data acquisition; however, its spatial resolution is limited by detector array density, and the overall cost remains relatively high.

The three-dimensional point cloud data produced by LiDAR directly capture the geometric features of the canopy, such as height, width, branching hierarchy, porosity, and total volume, thereby supporting the construction of high-precision canopy structure models [42,43]. Moreover, LiDAR sensing is independent of ambient illumination, making it robust under shaded, low-light, or nighttime conditions—an essential advantage for orchards with complex and uneven lighting. LiDAR’s ability to perform continuous sampling at different height levels also supports multiscale modeling requirements across various orchard configurations, including trellis systems and free-standing trees.

### 2.2. Vision Sensors

Vision sensors, which rely on image acquisition, are designed to capture information related to color, shape, texture, and other surface features of target objects. Owing to their low cost, compact structure, and flexible deployment, they serve as essential tools for canopy recognition and structural perception in orchard environments. Based on their imaging configuration and spatial information acquisition capabilities, vision sensors can be categorized into conventional monocular or stereo (multi-view) systems and RGB-D systems that integrate depth information. Each type offers unique advantages and can be selected according to canopy complexity and the type of agricultural platform in use.

#### 2.2.1. Monocular and Stereo (Multi-View) Vision Systems

Monocular vision systems, consisting of a single camera, are capable of capturing high-resolution two-dimensional (2D) image data. As the simplest and most widely deployed visual perception approach, they offer ease of implementation and broad applicability. The representative performance parameters of typical monocular devices are summarized in Table 2. These systems perform well in extracting surface-level canopy features such as texture, color, and edge contours, making them suitable for low-cost image acquisition scenarios [44,45,46,47]. However, due to their inherent lack of depth perception, spatial information must be inferred through image sequence modeling or learned representations. This reliance on algorithmic inference, coupled with sensitivity to ambient lighting conditions, can limit the accuracy of three-dimensional (3D) reconstruction. 

Stereo or multi-view vision systems (e.g., binocular or trinocular cameras) perceive canopy structure by computing depth information from image disparities between two or more viewpoints. These systems can generate either dense or sparse depth maps, which are useful for estimating the local morphology and spatial distribution of orchard canopies [48,49,50,51,52,53]. Compared to LiDAR, stereo vision systems offer lower cost and the ability to simultaneously capture both color and geometric information, making them well-suited for static or close-range operations. Typical applications include structural interpretation in regions with overlapping branches and leaves, as well as analysis of hierarchical relationships among foliage layers.

However, the performance of stereo vision systems is highly sensitive to ambient lighting conditions and surface texture features. In orchard environments, where leaves and fruits often exhibit high reflectivity and limited texture variation, stereo matching errors are common and can lead to inaccurate depth estimation. Moreover, these systems require precise camera calibration, and any deviation caused by mounting position shifts or vibrations can adversely affect imaging quality and modeling accuracy.

#### 2.2.2. RGB-D Vision Sensors

RGB-D vision systems extend traditional RGB imaging by incorporating an additional depth channel, enabling simultaneous perception of both color and three-dimensional (3D) structural information of target objects [54,55,56,57]. Representative performance specifications of commonly used RGB-D devices are presented in Table 3. Typical RGB-D systems acquire depth information through techniques such as structured light, time-of-flight (ToF), or active stereo vision. Compared with multi-view stereo systems, RGB-D systems offer higher structural integration and produce unified data formats, making them particularly suitable for mobile or embedded perception platforms.

Structured light cameras (e.g., Kinect V1) estimate depth by analyzing the deformation of projected dot patterns, offering high accuracy in close-range and static scenes. Time-of-flight (ToF) cameras (e.g., RealSense D455) calculate distance based on the flight time of light pulses, featuring high frame rates and strong stability, making them well-suited for capturing dynamic targets in orchard environments. Active stereo systems combine infrared projection with binocular disparity, providing a balance between measurement accuracy and environmental adaptability

By aligning the depth map with the corresponding RGB image, RGB-D systems can generate colored point clouds that capture the geometric structure of the tree canopy, leaf distribution, and spatial porosity [58,59,60,61]. These systems are capable of operating reliably in low-light or partially occluded environments, making them ideal for close- to mid-range canopy perception tasks in trellised orchards or low-growing shrubs. Due to their compact design and high computational efficiency, RGB-D cameras are increasingly adopted as standard visual modules in automated spraying robots and intelligent perception systems.

However, the effective depth-sensing range of RGB-D systems is typically limited to 0.3–5 m, which constrains their ability to capture the full canopy structure of tall orchard trees. In addition, the depth accuracy of these systems is susceptible to interference from ambient sunlight—particularly under strong illumination—where infrared projection can be severely compromised, resulting in noisy or incomplete depth maps. Current research efforts are primarily focused on enhancing depth image quality, extending sensing range, and improving robustness under varying environmental conditions.

### 2.3. Multispectral and Hyperspectral Sensors

Multispectral and hyperspectral imaging sensors are capable of capturing reflectance information across multiple spectral bands, enabling detailed analysis of object materials, physiological status, and spectral characteristics [62]. Representative performance specifications of commonly used devices are summarized in Table 4. Unlike conventional RGB imaging, these sensors not only acquire color and morphological features but also provide fine-grained spectral characterization, which supports a wide range of agricultural applications such as crop classification, pest and disease diagnosis, and nutrient assessment. In recent years, such sensors have also been increasingly employed for canopy structure perception in fruit orchards [63,64,65,66].

Multispectral imaging systems typically capture 3–10 discrete spectral bands spanning the visible to near-infrared regions and are well-suited for routine analysis of canopy features. In contrast, hyperspectral systems acquire tens to hundreds of contiguous spectral bands, offering higher spectral resolution and enabling the detection of subtle physiological and structural variations within the canopy. Both multispectral and hyperspectral sensors are commonly implemented using push-broom scanning, filter-based arrays, or imaging spectrometers, respectively, adapted for aerial inspection, ground-based monitoring, and high-precision static sensing.

In orchard environments, fruit tree canopies are characterized by multiple overlapping layers of branches and leaves, interspersed with irregular gaps, resulting in complex and heterogeneous structures. Multispectral and hyperspectral sensors can exploit reflectance differences across bands to differentiate leaf age, water content, and thickness—parameters that influence spectral responses. This capability supports canopy stratification and structural inference [67,68,69,70,71]. For instance, red-edge and near-infrared bands are particularly effective in distinguishing healthy from senescent foliage, serving as complementary indicators for spatial modeling and physiological monitoring [72,73,74]. Moreover, canopy gaps typically exhibit low reflectance across multiple wavelengths, which facilitates the estimation of canopy porosity.

Despite their rich spectral information, spectral imaging systems face several limitations, including large data volumes, limited real-time processing capabilities, sensitivity to ambient illumination, high device costs, and complex integration requirements. As a result, their current application is primarily restricted to research platforms and high-end sensing systems. In recent years, the development of lightweight devices such as the MicaSense Red Edge and Parrot Sequoia has enabled the gradual integration of spectral sensors into orchard-based unmanned aerial vehicles (UAVs) and ground platforms. The fusion of spectral data with RGB and LiDAR information for structure–spectrum collaborative perception has emerged as a promising direction to enhance modeling accuracy and semantic interpretation.

In summary, multispectral and hyperspectral imaging sensors serve as valuable tools for supplementing structural analysis and identifying spectral features within orchard canopies. They are particularly effective in capturing subtle canopy variations, extracting non-structural information, and supporting the development of integrated structural–physiological models, positioning them as key components of future high-precision orchard sensing systems.

## 3. Canopy Perception Technologies

### 3.1. LiDAR-Based Canopy Perception

Owing to its superior capability in spatial structure analysis and strong resistance to ambient light interference, LiDAR has become one of the core sensing technologies for three-dimensional canopy modeling in orchard environments. By actively emitting laser pulses and measuring their round-trip time, LiDAR generates high-precision point cloud data that capture the geometric characteristics of tree canopies, including height, crown width, hierarchical structure, and porosity. These spatial features are of critical importance in variable-rate spraying systems, particularly for identifying target regions and regulating pesticide dosage with precision.

**Figure 1:**
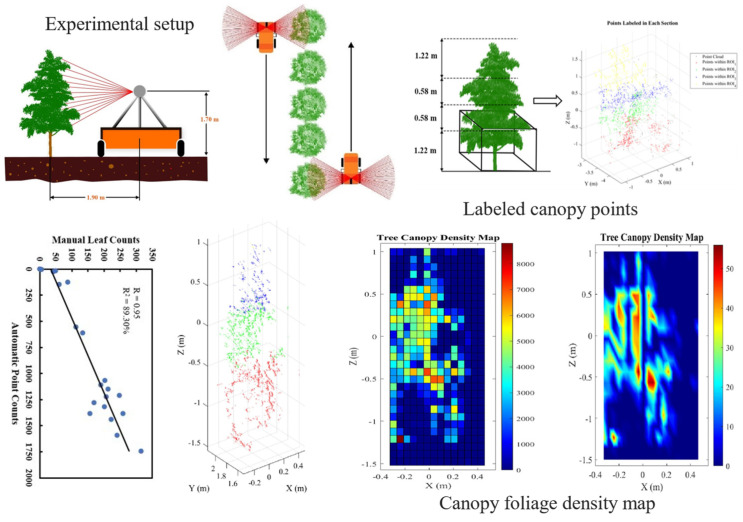
Workflow of LiDAR-based canopy density reconstruction and validation: In this study, 3D point clouds of fruit trees were acquired using a LiDAR sensor. A stratification method was applied to label different canopy zones, followed by Euclidean clustering to separate leaf and branch structures. A canopy density heatmap was generated to visualize the spatial distribution of point clouds. Validation using manual leaf counts confirmed a strong correlation between point cloud density and actual leaf quantity (R^2^ = 0.9056), providing structural insights for supporting variable-rate spraying strategies.

Current research extensively employs multi-line LiDAR sensors mounted on ground vehicles or unmanned aerial platforms to scan and model the canopies of fruit trees such as apple and citrus. Mahmud et al. [75] proposed a point cloud reconstruction method for fruit trees using a 16-line LiDAR system. The approach utilized Euclidean clustering to segment trunk and foliage components, followed by region growing and structural extraction algorithms to reconstruct the three-dimensional topological structure of apple trees, as illustrated in Figure 1. Experimental results demonstrated that the method achieved high segmentation accuracy and strong structural fidelity, even under complex canopy configurations. The reconstruction error was maintained within ±5 cm, providing reliable support for canopy density classification and the development of differentiated spraying strategies. A summary of representative LiDAR-based canopy perception techniques is presented in Table 5.

In terms of technical optimization, researchers have introduced inertial measurement units (IMUs) to enhance modeling stability and robustness by enabling pose correction and time synchronization. This approach effectively mitigates point cloud distortion caused by platform vibrations or terrain undulations. Meanwhile, with the advancement of deep learning in 3D data processing, point cloud neural networks such as PointNet++ have been applied to achieve high-precision branch recognition and structural segmentation [80]. To address the issue of severe occlusion in LiDAR-based sensing, multi-view point cloud stitching strategies have been proposed to recover occluded structural information [81]. A summary of canopy perception optimization techniques is provided in Table 6.

Nevertheless, several challenges remain in the application of LiDAR systems in orchard environments: the relatively high cost of LiDAR devices limits their large-scale deployment in small- and medium-sized orchards; leaf motion caused by wind disturbances can result in point cloud artifacts, including ghosting, data loss, and spatial voids, thereby affecting modeling accuracy; and the ability to sense and reconstruct occluded regions is limited—particularly in areas with overlapping branches and leaves, fruit obstructions, or deep canopy layers—leading to significant point cloud incompleteness.

To address these occlusion-related issues, recent studies have proposed a variety of solutions, such as multi-view and multi-frame data fusion, point cloud completion algorithms based on structural priors, and point cloud reconstruction using image guidance or deep learning inference models. Future research may further explore lightweight multi-sensor fusion schemes—such as integrating LiDAR with RGB or time-of-flight (ToF) cameras—combined with real-time point cloud enhancement networks to improve structural recovery and data availability in occluded regions. Such advancements would contribute to the development of high-precision and efficiently integrated perception modules for variable-rate spraying systems.

In summary, LiDAR remains a core sensing technology for canopy structural modeling, offering unique advantages in complex, unevenly illuminated, and long-range orchard scenarios. When paired with advanced perception algorithms and multimodal data fusion strategies, LiDAR holds significant promise for enabling high-precision, multiscale, and dynamic canopy sensing in future orchard management systems.

### 3.2. Visual and Multispectral-Based Canopy Perception

Visual imaging systems—particularly those utilizing RGB and multispectral imagery—have received increasing attention in recent years for canopy perception in orchard environments. These systems offer notable advantages, including low hardware cost, rich information content, and flexible acquisition methods. They are particularly well-suited for identifying canopy appearance characteristics such as morphology, color, texture, and signs of pests or diseases [86,87,88,89]. Current research in this domain generally follows two main directions: image-based structural recognition and spectral-based physiological state monitoring.

**Figure 2:**
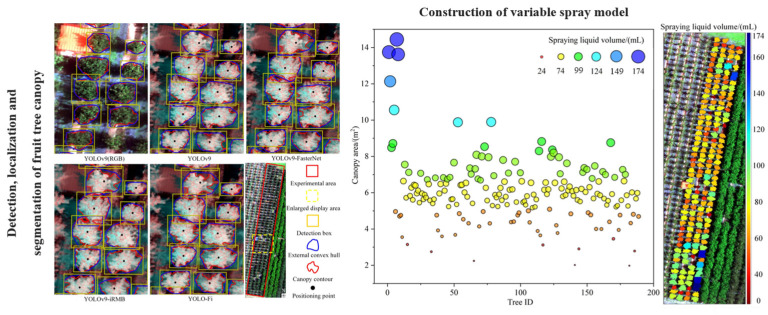
Deep learning-based fruit tree canopy segmentation and variable-rate spraying model: The figure illustrates the process of fruit tree canopy detection, localization, and segmentation using UAV-acquired remote sensing imagery and the YOLO-Fi model (left), the construction of a variable-rate spraying model based on canopy area estimation (center), and the spatial visualization of orchard spraying operations (right). This method enables precise matching between canopy features and spray volume, providing a complete perception-to-decision workflow for intelligent orchard management. Colored circles in the center panel represent different canopy-level spray volumes, increasing progressively from red to dark blue, corresponding to approximately 24 mL, 74 mL, 99 mL, 124 mL, 149 mL, and 174 mL, respectively.

In terms of structural recognition, deep convolutional neural networks such as YOLO, Mask R-CNN, and UNet [90,91,92,93,94] have been widely applied for fruit detection, leaf segmentation, and tree shape feature extraction [95,96,97]. When integrated with mobile platforms in orchard environments, these systems can acquire and analyze canopy images in real time to extract spray zone boundaries and identify fruit-dense regions. Wei et al. developed a spray-target identification model based on YOLO-Fi, which enabled fruit tree detection, localization, and canopy segmentation [98]. The model demonstrated strong generalization performance, achieving a mean average precision of *mAP*_50–95_ = 0.862. As shown in Figure 2, the system was capable of effectively segmenting canopy regions and generating variable-rate spray prescription maps. A summary of vision-based canopy perception techniques is provided in Table 7.

**Figure 3:**
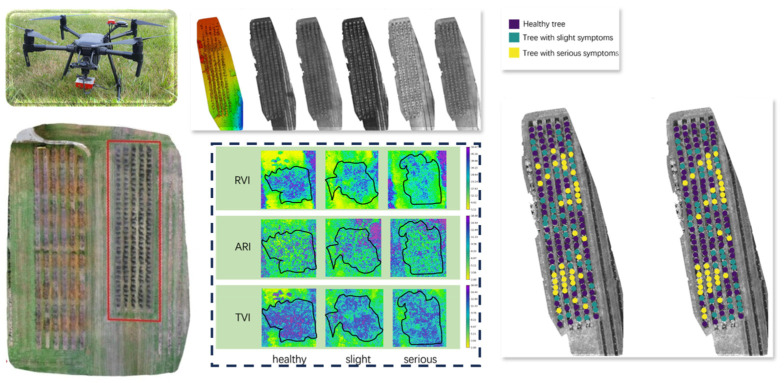
UAV-based multispectral remote sensing workflow for apple fire blight detection: This figure illustrates the use of a UAV platform equipped with a multispectral camera to acquire orchard imagery, including multi-band orthomosaics of the target area. It presents the spectral response characteristics of typical vegetation indices (RVI, ARI, and TVI) across apple trees with varying levels of disease severity and shows the spatial distribution of fire blight identified using an optimal feature set and a random forest classification model. The results validate the feasibility and discriminative power of multispectral vegetation indices for early-stage disease diagnosis in fruit trees.

In terms of physiological state analysis, multispectral imaging enables the acquisition of data across multiple spectral channels, including red-edge and near-infrared bands. By calculating vegetation indices—such as the Normalized Difference Vegetation Index (NDVI), Modified Chlorophyll Absorption Ratio Index (MCARI), and Photochemical Reflectance Index (PRI)—canopy chlorophyll content, water stress, and early-stage disease symptoms can be effectively assessed. These metrics are particularly useful for regulating pesticide concentration and implementing localized disease-targeted spraying strategies in variable-rate spraying systems. A recent study developed a fire blight detection model for apple orchards using UAV-based multispectral imagery combined with an optimal set of vegetation indices (RVI, ARI, and TVI). The model employed a random forest classifier and achieved a high classification accuracy of 94.0%, demonstrating the feasibility and effectiveness of multispectral remote sensing in early disease detection for fruit trees [105], as illustrated in Figure 3.

Hyperspectral systems offer even finer differentiation of canopy-level micro-variations across hundreds of spectral bands. However, their high equipment cost and complex data processing requirements currently limit their deployment frequency in field operations. A summary of multispectral-based canopy perception technologies is provided in Table 8.

Despite the widespread application of image-based systems, several technical bottlenecks remain as follows: image acquisition is highly sensitive to ambient lighting conditions, with shadows and high-contrast regions significantly degrading recognition accuracy; the lack of structural depth information limits the ability to model canopy spatial distribution and occlusion relationships; and under wind-induced motion, image continuity is disrupted, reducing the robustness of dynamic object recognition.

To address these limitations, some studies have explored the integration of structured light or stereo vision technologies. However, their performance in complex canopy environments remains constrained by disparity range and system stability. Researchers are actively developing more robust visual processing frameworks. These include incorporating Transformer architectures to enhance spatial modeling capabilities, employing image style transfer to improve generalization under varying lighting conditions, and implementing image–point cloud co-annotation strategies to improve semantic consistency. In addition, lightweight vision models—such as MobileNet and EfficientNet—are being deployed on edge computing platforms, enabling real-time perception support for variable-rate spraying operations in field conditions.

Overall, visual and multispectral imaging systems provide efficient information on canopy appearance and health status. However, to achieve comprehensive canopy perception, these systems must be integrated with complementary sensors in order to construct joint models of structural and physiological attributes.

### 3.3. Canopy Perception Based on Multi-Source Data Fusion

In orchard environments, a single sensing modality often fails to simultaneously satisfy the diverse requirements of structural modeling, physiological status assessment, and dynamic adaptability. As a result, multimodal sensor fusion has emerged as a promising approach to enhance canopy perception capabilities. The core principle is to integrate heterogeneous sensors—such as LiDAR, RGB cameras, multispectral/hyperspectral imagers, depth cameras, and environmental sensors—to achieve information complementarity, redundancy reduction, and decision-level coordination [112,113].

**Figure 4:**
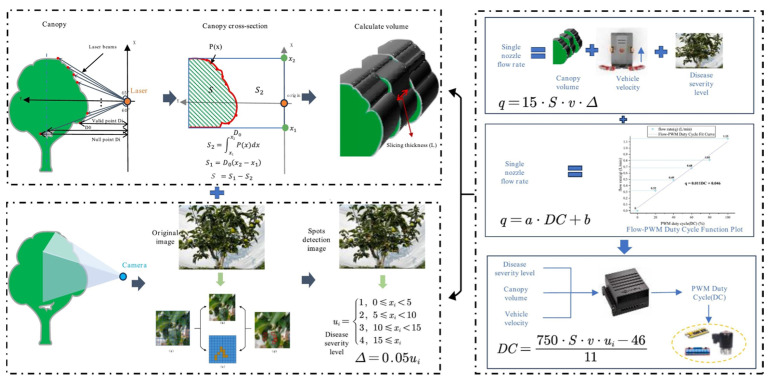
Conceptual framework of the variable-rate spraying strategy based on fused canopy volume and disease severity information: The upper-left box shows canopy volume estimation using laser scanning and polynomial-based cross-sectional fitting. The lower-left box presents disease detection via YOLOv5 to determine severity levels (*u*_i_) and corresponding unit spray volumes (Δ = 0.05*u*_i_). The right-hand box integrates these inputs to calculate nozzle flow rate (*q*) and PWM duty cycle (*DC*), enabling real-time spray adjustment based on canopy and disease conditions.

LiDAR enables point cloud reconstruction of the orchard environment, providing detailed spatial structure information of fruit trees, including canopy volume, leaf density, and branch distribution. Meanwhile, multispectral sensors capture spectral reflectance in various bands, offering insights into physiological parameters such as chlorophyll content and early-stage disease symptoms. By registering point cloud data with spectral imagery, it becomes possible to jointly analyze canopy morphology and crop health. Zhang et al. [114] proposed a feature-level fusion-based variable-rate spraying approach that integrates canopy volume characteristics and disease spot information at the perception layer. By combining YOLOv5 with LiDAR, the system enables synchronized acquisition of disease severity and structural volume data, facilitating a unified perception-control framework for dynamically optimized spray decision-making. Experimental results demonstrate that this method outperforms conventional single-indicator strategies in both chemical usage reduction and disease suppression, effectively balancing plant protection efficacy and resource efficiency, as illustrated in Figure 4.

Furthermore, the integration of LiDAR with the vision-based YOLOv5 model leverages the complementary strengths of each sensor modality—namely, the deep-penetrating structural perception capability of LiDAR and the high-precision disease localization ability of YOLOv5. This synergy makes the approach particularly suitable for variable-rate spraying tasks that require simultaneous attention to canopy structure and plant health status.

Visual sensors capture information in the visible spectrum, including color and texture, and are well-suited for target recognition and localization. However, they are limited in their ability to reflect physiological attributes of crops. In contrast, multispectral sensors can detect plant health, water stress, and pest infestation across multiple spectral bands, but often suffer from lower spatial resolution and weak structural representation. The fusion of these two modalities combines the detailed spatial expression of visual imagery with the spectral sensitivity of multispectral data, thereby enhancing the accuracy and comprehensiveness of orchard target identification and crop status assessment. For example, Li et al. [115] proposed a data-level fusion approach in which RGB images and multispectral vegetation indices were concatenated at the channel level. The model incorporated the ReliefF algorithm and a channel attention mechanism to enhance sensitivity to disease- and pest-related features. Results showed that the proposed AMMFNet fusion model significantly improved the diagnostic accuracy and robustness for orchard pest and disease detection.

However, the deployment of fusion systems faces several practical challenges: Cross-sensor calibration is complex and susceptible to vibration and temperature drift; high requirements for data synchronization demand real-time acquisition and processing at high frame rates; and fusion algorithms are often computationally intensive, making deployment on low-power devices difficult. Moreover, the differences in data quality and noise characteristics across sensing modalities pose additional challenges for achieving robust integration.

To address these issues, current research trends focus on three main directions: First, developing lightweight cross-modal feature extraction models to support edge computing deployment. Second, introducing adaptive fusion mechanisms that dynamically adjust sensor weighting based on environmental conditions. Third, exploring hybrid frameworks that integrate physical priors with deep learning to enable dynamic coordination in perception–control closed-loop systems.

In summary, multimodal fusion technologies not only enhance the overall performance and adaptability of perception systems but also provide high-dimensional, dynamic, and scalable sensing support for intelligent decision-making in variable-rate spraying systems. As such, they represent a critical direction in the development of advanced perception systems for smart agricultural machinery.

## 4. Application of Canopy Perception Technologies in Variable-Rate Spraying Systems

### 4.1. Spray Zone Partitioning and Nozzle Control Strategies

In variable-rate spraying (VRS) systems, the primary objective of spray path planning and nozzle control strategies is to achieve “spray as needed” and “minimal overlap redundancy.” This entails dynamically generating spray trajectories and configuring nozzle activation and spraying parameters based on the structural characteristics and physiological status of the target canopy. The ultimate goal is to improve pesticide utilization efficiency and spray uniformity.

Canopy perception models play a critical role in this process, particularly in spatial localization of spray targets, canopy density-based zone partitioning, and prioritization of spray regions. These models enable the system to distinguish between areas requiring intensive treatment and those needing minimal or no spraying, thereby supporting precise, data-driven control decisions.

**Figure 5:**
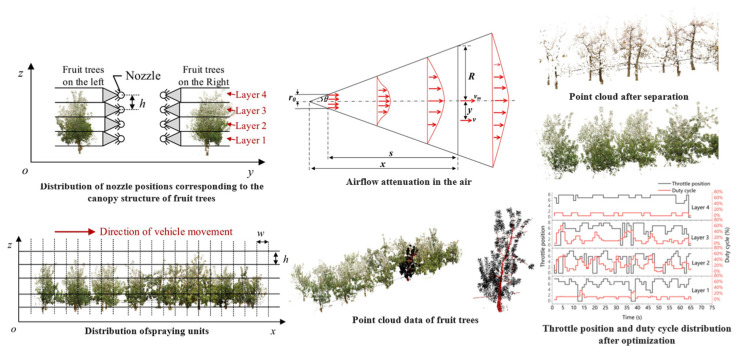
Orchard variable-rate spraying driven by multidimensional prescription maps: This figure illustrates the key technical workflow for constructing multidimensional spray prescription maps based on real fruit tree point cloud data. The process includes nozzle layer structuring, wind speed attenuation modeling, leaf–wood segmentation, spray unit partitioning, and control parameter optimization, providing a systematic approach for achieving precision variable-rate spraying. Specifically, nozzle layer structuring means arranging nozzles in vertical layers aligned with canopy heights, enabling accurate spray delivery and reduced drift.

Early approaches primarily relied on static LiDAR-based canopy models to generate spray unit partitioning, typically applying uniform-speed and fixed-interval spraying patterns. More recent studies have incorporated real-time canopy perception systems that leverage point cloud density, fruit distribution, and disease lesion locations to implement multi-level priority spraying via weighted coverage map generation algorithms. For example, Hu et al. [116] proposed an orchard variable-rate spraying method based on multidimensional prescription maps. The method integrates point cloud-based leaf–wood segmentation, nozzle topology optimization, and models for wind-speed-aware droplet delivery and dosage control. Its effectiveness and precision were validated through hardware-in-the-loop (HIL) simulations and field experiments, as illustrated in Figure 5. A summary of canopy perception-driven variable-rate spraying techniques is provided in Table 9.

**Figure 6:**
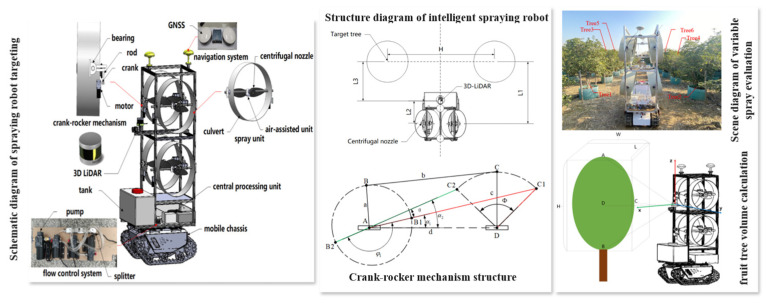
Schematic of the 3D-LiDAR-based multivariable spraying robot and its control mechanism: The robot integrates 3D LiDAR sensing, PWM-controlled pumps and nozzles, air-assisted spray units, and an articulated arm to achieve full-parameter control of spray rate, airflow intensity, droplet size, and spray direction. Left: Overall system architecture, including sensing, control, and execution modules. Center: Target localization, canopy volume estimation, and the operation of the articulated spray boom. Right: Field experiment demonstrating variable-rate spraying guided by canopy structure data.

In terms of nozzle control, variable-rate spraying systems typically employ electromagnetic or PWM-regulated nozzles to adjust multiple spray parameters, including on–off status, spray cone angle, pressure level, and droplet size. The canopy distribution information obtained from the perception model is transmitted in real time to the control unit, which drives the nozzles to operate precisely at optimal timings. Liu et al. [123] developed a multi-parameter spraying system centered on 3D LiDAR perception. The system enables full-parameter regulation—including spray flow rate, airflow intensity, droplet size, and spray direction—based on the spatial distribution of the target canopy, as shown in Figure 6. The control module employs Pulse-Width Modulation (PWM) signals to dynamically regulate electromagnetic valves and centrifugal nozzles, ensuring timely and accurate activation of each nozzle. This approach facilitates efficient pesticide savings and uniform droplet deposition.

However, current approaches to spray path planning and nozzle control still face three major challenges: path planning is typically based on static canopy models, lacking adaptability to dynamic disturbances such as wind-induced motion and occlusions; nozzle control granularity is limited, making it difficult to achieve fine-grained, multi-level regulation in response to complex target structures; and there is no clearly defined prioritization mechanism for competing targets—such as fruits and disease lesions—and few existing methods incorporate optimization algorithms to balance these objectives.

Future development should focus on three key directions: integrating temporal perception outputs and adopting reinforcement learning strategies (e.g., Deep Q-Networks) to enable dynamic path generation under changing conditions; establishing a closed-loop “target–control–response” framework by coupling spray simulation models with real-time perception feedback; and developing multivariable control systems capable of spatiotemporal coordination of spray intensity, frequency, and direction, enabling full-dimensional, adaptive control for complex orchard scenarios.

### 4.2. Boom Attitude Adjustment and Dynamic Response Mechanisms

As the direct interface between the spraying system and the target crop canopy, the spatial posture of the spray boom has a critical impact on spray quality. In orchard environments, canopy height varies considerably, and terrain undulations between tree rows are often complex. If the boom cannot dynamically adjust its pitch angle and vertical position in real time, it may result in under-coverage or redundant respraying, thereby reducing pesticide use efficiency and compromising operational safety. Structural gradient and height information provided by canopy perception models offers essential input for enabling dynamic boom adjustment.

**Figure 7:**
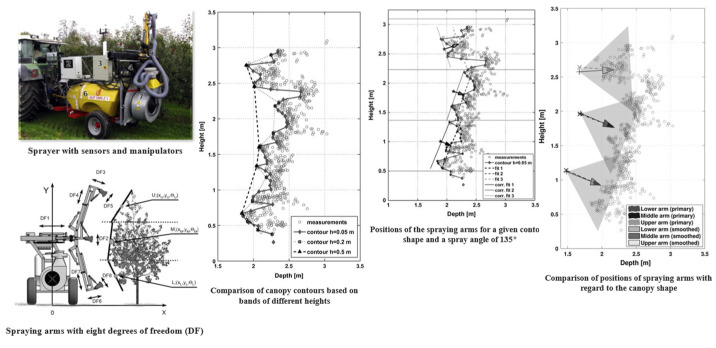
LiDAR-based real-time boom adjustment system and canopy-tracking method for orchard spraying: This figure presents the architecture and core functions of a LiDAR-based boom posture control system. A front-mounted LiDAR sensor captures the canopy’s spatial profile, which is vertically stratified to extract characteristic layers. A 0.05 m bandwidth yields a more accurate canopy reconstruction. Based on canopy contours and spray geometry, the system computes optimal nozzle orientation and distance for each layer, enabling real-time height and pitch adjustment of the boom.

Traditional boom control strategies often rely on fixed-height configurations or passive mechanical float systems, which are insufficient for adapting to spatially heterogeneous canopy structures. These systems rely on springs or linkages to passively adjust boom height in response to ground surface variations, but they cannot respond effectively to complex canopy topography. Recent advancements have introduced localized height-sensing systems based on LiDAR or ultrasonic sensors, which enable real-time measurement of the distance between the boom and the canopy top. These measurements are used to drive electric or hydraulic servo systems for adjusting both the boom’s angle and height. Osterman et al. [124] proposed a LiDAR-based boom posture control method that reconstructs real-time canopy profiles to calculate local surface inclinations at different height levels. By incorporating spray angle geometry, the system determines the optimal nozzle orientation and distance, as illustrated in Figure 7. A hydraulic actuation mechanism enables synchronized adjustment of the boom’s height and angle within the vertical plane, allowing the system to accommodate spatially uneven canopy architectures. Compared to traditional fixed or mechanically floating systems, this method demonstrates superior performance in spray accuracy and operational adaptability.

This study developed an integrated “perception–computation–control” feedforward regulation model to enable real-time alignment of nozzle orientation with the fruit tree canopy profile. The core process is as follows:

(1) $$\left\{\begin{array}{l} D_{n}=[X_{n},\;d_{1},\;d_{2},\;\ldots ,\;d_{8}]\\ \varphi_{n}=[X_{n},\;\alpha_{1},\;\alpha_{2},\;\alpha_{3}]\\ P\text{\_}s\;=\;X\text{\_}n\;-\;L \end{array}\right.$$

In the equation, *D_n_* represents the canopy distance vector acquired in real time when the sprayer is at position *X_n_*; *φ_n_* denotes the computed posture angles of the three-section spray boom; and P_s introduces the fixed offset L between the sensor and the nozzle to achieve spatial compensation of the spray position.

By continuously solving canopy profile information in real time, the model dynamically adjusts nozzle orientation to match canopy surface variations, thereby enhancing spray coverage conformity and deposition efficiency. Although the current control strategy does not yet account for wind disturbances or canopy dynamic responses, this perception-to-posture mapping framework establishes a solid foundation for the future development of wind–canopy–spray coupled control systems

Beyond vertical height adjustment, spray boom posture also includes pitch angle, swing amplitude, and structural compliance. Some studies have explored the development of coupled boom–crop dynamic models, in which canopy structural information obtained through perception is fed into the control loop. Model predictive control (MPC) frameworks are then employed to adjust boom trajectories in anticipation of canopy variations.

**Figure 8:**
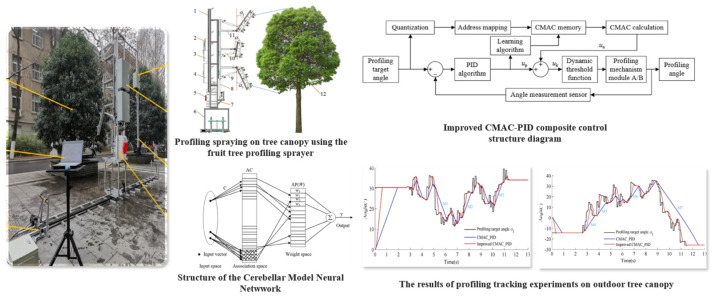
Structure of the boom attitude control system and experimental results of tracking performance: This figure presents the structure and field validation of an intelligent boom attitude adjustment control system based on canopy contour tracking. The system integrates an ultrasonic sensing module, an improved CMAC-PID composite controller, and a multi-degree-of-freedom boom actuation mechanism. It dynamically adjusts nozzle orientation according to the canopy inclination, enabling precise variable-rate spraying. Field experiments confirmed the system’s excellent response speed and tracking accuracy.

For example, Nan et al. [125] designed a boom attitude tracking control system for orchard sprayers based on canopy phenotypic features, as illustrated in Figure 8. The system integrates ultrasonic sensing with an enhanced CMAC-PID (Cerebellar Model Articulation Controller–Proportional Integral Derivative) control algorithm, enabling real-time adjustment of joint angles across a multi-segment boom (segments *A_up_*, *B*, and *A_down_*) to conform to canopy contours. Experimental results demonstrated significant improvements in response speed and tracking accuracy, validating the effectiveness of the proposed method for dynamic boom posture adjustment in variable-rate spraying operations.

Despite recent advances, several challenges remain in this field: conventional boom structures often lack sufficient rigidity, and frequent posture adjustments may lead to mechanical resonance or structural fatigue; the spatial accuracy of canopy models is significantly affected by occlusions and wind-induced movement, increasing the likelihood of misjudgment; and the response time of boom adjustment algorithms often fails to match the operational speed of field machinery, compromising overall system stability.

Future research directions are recommended as follows: develop lightweight, flexible boom structures using advanced composite materials to improve adaptability and reduce mechanical stress; integrate redundant multi-sensor configurations to enhance the robustness of canopy modeling under complex field conditions; and introduce feedforward control mechanisms and deep reinforcement learning strategies to achieve faster, more accurate dynamic boom posture control.

### 4.3. Real-Time Feedback and Closed-Loop Coordinated Control

In addition to high-precision perception and modeling capabilities, variable-rate spraying (VRS) systems must incorporate a closed-loop control framework encompassing perception, decision-making, execution, and feedback. Real-time feedback systems continuously acquire response data during spraying operations—such as droplet deposition, nozzle status, and wind speed variations—and compare these with model predictions to dynamically adjust spray strategies. This mechanism enhances operational stability and system intelligence.

Feedback data acquisition can be broadly categorized into two types: structural feedback (e.g., canopy variation) and outcome feedback (e.g., deposition quality). Structural feedback is typically obtained through real-time LiDAR scanning, RGB video streams, and IMU-based posture fusion, and is primarily used to detect canopy occlusion changes and leaf vibration patterns. In contrast, outcome feedback relies more on post-operation assessment methods, such as droplet deposition sensors, water-sensitive paper analysis, high-speed imaging, or fluorescent tracer techniques. These approaches are not yet capable of supporting real-time control but can still serve as valuable empirical data sources to identify under-sprayed areas. This information can be used to guide subsequent operations with enhanced coverage and parameter optimization, thereby indirectly improving spray uniformity and deposition quality.

**Figure 9:**
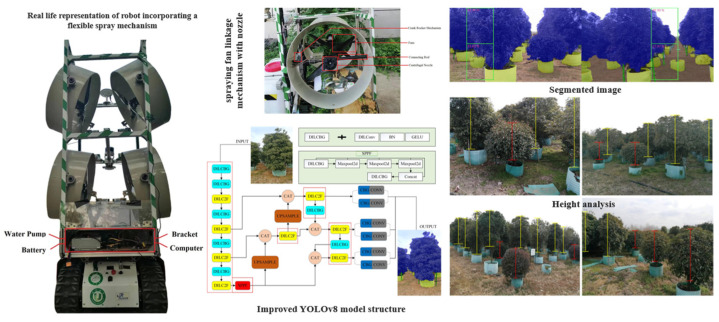
Orchard spraying robot and spray mechanism based on the improved YOLOv8 algorithm: The left panel shows a spraying robot equipped with a flexible spray mechanism, while the right panel presents real-time orchard canopy segmentation results using the improved YOLOv8 algorithm. The system enables accurate segmentation of target areas, significantly enhancing the precision and efficiency of pesticide application. By performing instance segmentation in real time, the system reduces non-target spraying, optimizes droplet deposition, and improves overall pesticide utilization efficiency.

Khan et al. [126] proposed an enhanced YOLOv8-based algorithm for real-time, high-precision segmentation of orchard canopies, aiming to accurately identify target areas and optimize pesticide application accuracy and efficiency, as illustrated in Figure 9. Experimental results showed that the variable-rate spraying system employing this algorithm reduced non-target spraying by 40.39% compared to conventional methods, significantly improving spray precision and pesticide use efficiency. A summary of real-time feedback mechanisms and closed-loop coordination control technologies is provided in Table 10.

At the control system level, some advanced VRS platforms have adopted bidirectional communication architectures that integrate the perception, control, and actuation modules into a coordinated system. These systems can dynamically adjust vehicle speed and nozzle flow rate in response to environmental variations during spraying, effectively mitigating pesticide drift caused by wind disturbances. In addition, perception–control systems can iteratively optimize spray trajectories based on droplet deposition feedback, ultimately achieving precise coverage of target areas.

However, closed-loop systems have not yet been widely implemented in practice, primarily due to the following limitations: high-frequency data acquisition and processing place substantial computational demands on edge devices; real-time monitoring of droplet deposition remains technically challenging, particularly within internal canopy regions; and the high degree of coupling between system modules reduces fault tolerance and makes the system more susceptible to disturbances.

Future research is encouraged to focus on the following directions: develop lightweight feedback control systems based on edge computing to enhance real-time responsiveness; integrate large-scale virtual training environments by coupling perception systems with spray-effect simulation platforms; and combine closed-loop control frameworks with dynamic modeling approaches—such as Physics-Informed Neural Networks (PINNs) and hybrid temporal networks—to enable the transition from offline calibration to online adaptive intelligence.

## 5. Current Challenges and Future Directions

### 5.1. Key Challenges

Perception and modeling in orchard variable-rate spraying systems still face several critical bottlenecks, which severely limit their stability and scalability under complex field conditions.

Trade-off between real-time performance and modeling accuracy: While high-resolution point clouds and hyperspectral images can provide detailed structural and physiological information, their processing speed is often too slow to support real-time decision-making during spraying operations. On the other hand, lightweight models offer faster response times but typically suffer from limited accuracy, particularly in complex canopy structures or under dynamic occlusion scenarios.

Lack of model adaptability: Orchard canopies vary significantly across seasons, tree varieties, and management practices. Most existing models are designed for specific scenarios and lack transfer learning capabilities. As a result, retraining is costly, and generalization to different orchard environments is limited.

Complex field disturbances and environmental variability: Orchard environments are subject to frequent disturbances such as fluctuating wind speeds, uneven terrain, and recurring occlusions, which impose stringent robustness requirements on perception and control systems. However, most existing systems exhibit limited disturbance tolerance and lack sufficient fault-handling mechanisms, often resulting in spraying errors or control failures. Given the substantial differences among canopy perception technologies in terms of cost, performance, and adaptability, Table 11 provides a comparative summary of key specifications and advantages for mainstream systems such as LiDAR, RGB-D, and multispectral sensors, thereby supporting informed decisions in system selection and deployment strategies.

Although LiDAR and multispectral sensors demonstrate superior structural modeling capabilities and adaptability to operational conditions, their hardware costs are considerably higher than those of conventional vision-based solutions—often 5 to 10 times that of stereo vision systems. This cost barrier has hindered large-scale deployment in small- and medium-sized orchards. Nonetheless, variable-rate spraying systems can achieve a 15–40% reduction in pesticide usage while improving pest and disease control effectiveness, leading to a typical return on investment within one to three growing seasons. For cost-sensitive applications, RGB-D systems at mid-range price points, when coupled with lightweight perception models, offer a cost-effective solution that balances budget constraints and sensing requirements, making them particularly suitable for small orchard operations.

In summary, advancing the practical deployment and engineering maturity of perception-modeling systems for variable-rate spraying requires addressing three critical trade-offs: real-time performance vs. accuracy, generalization vs. task-specific customization, and disturbance resilience vs. system stability.

### 5.2. Integration of Multiscale Modeling and Intelligent Control

Orchard spraying involves complex multiscale dynamic processes, including microscale interactions between droplets and leaf surfaces, mesoscale leaf cluster movement and occlusion, and macroscale flow coupling between canopy structures and wind fields. This hierarchical nesting implies that spray effectiveness is influenced by factors at multiple spatial and temporal scales, making it difficult for a single modeling framework to fully capture system dynamics.

Current mainstream approaches tend to adopt either physics-based simulations (e.g., CFD or FSI) or data-driven predictions using deep neural networks. While each has distinct advantages, both have notable limitations. Physics-based models offer high accuracy and strong interpretability but are computationally intensive, whereas deep learning models provide fast responses but often lack physical consistency and explanatory power.

As a result, future modeling trends are expected to converge toward physics-informed data-driven approaches. A representative example is the use of Physics-Informed Neural Networks (PINNs), which embed governing physical laws directly into the model training process, balancing computational efficiency with physical interpretability. In canopy wind response prediction tasks, PINNs have demonstrated superior generalization capability and convergence stability compared to conventional neural networks.

At the same time, intelligent control systems should be tightly integrated with perception and modeling components. Techniques such as deep reinforcement learning (DRL) can transform spray target recognition, path planning, and pressure adjustment into a joint optimization problem, enabling multi-objective variable-rate spraying control.

Looking ahead, next-generation spraying systems should embody a fully integrated “perception–modeling–control” architecture. By dynamically responding to multiscale canopy behavior, these systems will enable real-time strategy adaptation and achieve true closed-loop intelligent spraying.

### 5.3. Strategies for Developing Practical and Scalable Sensing Systems

Translating perception and modeling systems from laboratory settings to real-world orchard applications requires addressing three practical challenges: robustness, cost-effectiveness, and universality.

Enhancing system stability: A promising strategy is to implement multimodal redundant sensing architectures—such as LiDAR + RGB + IMU—combined with anomaly detection algorithms and state-aware mechanisms. These can enable self-diagnosis capabilities in spraying systems and improve operational resilience under field uncertainties.

Promoting system lightweighting and integration adaptability: Edge computing platforms—such as Jetson Nano and Xavier NX—can support lightweight perception models that have been pruned and optimized for real-time performance. When combined with low-power microcontrollers, these platforms enable the development of tightly integrated hardware–software collaborative systems. They offer favorable energy efficiency, with typical power consumption ranging from 5 to 15 W, and can operate continuously for 4–6 h on lithium battery packs, which is sufficient for a single orchard operation cycle. Furthermore, these devices support standard communication protocols such as ROS and CAN, ensuring strong compatibility and seamless integration with orchard field robots, including automated sprayers.

Improving model generalization and rapid adaptability: Incorporating incremental learning and transfer learning mechanisms allows perception models to rapidly adapt to different crop species and regional conditions by reconstructing structural features on the fly. Federated learning can further support cross-user collaborative optimization while preserving data privacy, thereby enhancing system universality across diverse operational environments.

In addition, the establishment of industry-wide standards should be prioritized. This includes developing open-access datasets, standardized evaluation protocols, and reusable software toolkits to lay the groundwork for large-scale dissemination of research outcomes and technology transfer into practice.

## 6. Conclusions

As the critical interface for droplet transport and pesticide efficacy in variable-rate spraying systems, orchard canopies play a decisive role in determining spray efficiency and pesticide utilization. This review systematically examined the key technological pathways for canopy perception and modeling in orchard environments. It focused on LiDAR-based sensing, visual and multispectral image processing, and multimodal data fusion strategies, highlighting the comparative capabilities of various data sources in structural reconstruction, physiological state recognition, and dynamic adaptation. Furthermore, the review organized mainstream canopy modeling approaches into three levels—static, quasi-dynamic, and fully dynamic—and provided a comprehensive comparison of their application scenarios, control system integration strategies, and engineering challenges within variable-rate spraying systems.

Although significant progress has been made in canopy modeling accuracy, multi-source data fusion, and closed-loop spray control, notable gaps remain in real-time performance, system robustness, and multiscale coupling capabilities—particularly under the complex and variable conditions of real-world orchard environments. Future research should prioritize the following: the integration of physics-based and data-driven modeling frameworks (e.g., PINNs); the development of multiscale canopy perception and decision-making systems; and the design of lightweight perception models that support edge deployment and generalizable applications.

It is worth noting that our research team has conducted a series of studies on orchard canopy perception and modeling. These efforts include the development of leaf tracking and detection models for quantifying the dynamic interaction between wind and foliage, as well as the integration of LiDAR and vision sensors to enable coordinated control between canopy structure perception and sprayer actuation. These practical achievements provide solid support for the key technological pathways and future research directions outlined in this review, while also demonstrating the feasibility and application potential of the proposed modeling strategies in real-world orchard scenarios.

This review begins with the integrated requirements of variable-rate spraying systems and systematically summarizes the current technological pathways for orchard canopy perception and modeling. It clarifies the applicable scenarios and evolutionary logic of different strategies, offering a structured overview that helps identify technical bottlenecks and integration challenges. The insights presented herein aim to guide future research efforts, promote the transition of perception technologies from prototype validation to engineering deployment, and enhance system intelligence and deployment efficiency.

## Figures and Tables

**Table 1:** Lidar performance parameters.

Sensor Model	Line Count	Range Capability	Accuracy	Frame Rate
Sick LMS111-10100 (SICK AG, Waldkirch, Germany)	1	0.5~20 m	±30 mm	25 Hz/50 Hz
RS-16 LiDAR (Robosense, Shenzhen, China)	16	0.4~150 m	±2 cm	5 Hz/10 Hz/20 Hz
Sick LMS511-20100 PRO (SICK AG, Waldkirch, Germany)	1	0.2~80 m	±12 mm	25 Hz/35 Hz/50 Hz/75 Hz/100 Hz
Helios 16 (Robosense, Shenzhen, China)	16	0.2~150 m	±1 cm	5 Hz/10 Hz/20 Hz

**Table 2:** Core performance specifications of representative monocular vision sensors.

Sensor Model	Shutter Type	Interface	Frame Rate	Resolution	Pixel Size
SHL 1600 4K (SHL/ShunhuaLi, Shenzhen, China)	Global	C/CS	60 fps	9280 × 5220	1.3 × 1.3 µm
MV CE200 10UM (Hikrobot, Hangzhou, China)	Rolling	USB 3.0	40 fps	5472 × 3648	2.4 × 2.4 μm
VLXN 490M.I.JP (Baumer Electric GmbH, Friedberg, Germany)	Global	5 GigE	18 fps	7008 × 7000	3.2 × 3.2 µm
VQXT 120C.HS (Baumer Electric GmbH, Friedberg, Germany)	Global	10G Ethernet	335 fps	4096 × 3068	5.5 × 5.5 µm

**Table 3:** Core performance specifications of representative RGB-D vision sensors.

Sensor Model	Depth Technology	Frame Rate	RGB Resolution	Depth Resolution
Femto Bolt (Orbbec, Shenzhen, China)	iToF (indirect ToF)	30 fps	320 × 288	1920 × 1080
Kinect v2 (Microsoft Corporation, Redmond, WA, USA)	ToF	30 fps	1920 × 1080	512 × 424
Intel RealSense D435i (Intel Corporation, Santa Clara, CA, USA)	Binocular Infrared	90 fps	1920 × 1080	1280 × 720
Femto Megal (Orbbec, Shenzhen, China)	ToF	30 fps	1920 × 1080	640 × 576

**Table 4:** Core performance specifications of representative multispectral cameras.

Sensor Model	Spectral Bands (MultiSpectral + PAN)	Sensor Resolution (Per Band)	FOV (H × V)	Storage Method	Power Supply
FS-500 (Focused Photonics, Hangzhou, China)	4× multispectral + 1× RGB	MS: 1.3 MP/RGB: 11.9 MP	MS: 69.1° × 56.4°/RGB: 59.1° × 45.0°	TF card	12 V DC
Red Edge-P (MicaSense, Seattle, WA, USA)	5× multispectral + 1× PAN	MS: 1.6 MP/PAN: 5.1 MP	50° HFOV × 38° VFOV	CF express	7–15.6 V DC
Altum-PT (MicaSense, Seattle, WA, USA)	5× multispectral + 1× PAN + thermal	MS: 3.2 MP/PAN: 12 MP	48° HFOV × 39° VFOV	CF express	7.0–25.2 V DC
AQ600 (Changguang Yuchen, Changchun, China)	5× multispectral (CMOS) + 1× RGB	MS: 3.2 MP/RGB: 12.3 MP	MS: 48.0° × 39.6°/RGB: 47.4° × 36.4°	Internal + SD/USB	12 V DC

**Table 5:** LiDAR-based canopy perception techniques.

Target Object	Detection Objective	Methodology	Ref.
Apple tree canopy	Leaf area estimation	3D point cloud segmentation	[76]
Pear tree canopy	Canopy volume/profile modeling	Grid-based contour extraction	[77]
Apple tree canopy	Leaf area estimation	Point cloud density and canopy volume correlation using regression model	[78]
Apple tree canopy	Canopy density estimation	Voxel-based occupancy analysis	[79]

**Table 6:** Optimization strategies for canopy perception.

Optimization Method	Detection Objective	Methodology/Technical Focus	Ref.
IMU-assisted pose correction	Accurate canopy point cloud acquisition and volume estimation	Sensor fusion for pose compensation	[82]
IMU-based slope evaluation	Canopy point correction and density estimation	Terrain-aware LiDAR data optimization	[83]
IMU-assisted pose optimization	Extraction of precise canopy structural features	Multi-frame alignment using IMU–LiDAR fusion	[84]
PointNet++-based segmentation	Measurement of canopy branch length	Deep learning-based 3D branch identification	[85]

**Table 7:** Vision-based canopy perception techniques.

Researcher	Research Object	Research Method	Research Objective	Ref.
Akdoğan et al.	Cherry and apple canopy	PP-YOLO classification model	Canopy classification and precision spraying	[99]
Sun et al.	Pear fruits	YOLO-P detection model	Accurate detection of pear fruits	[100]
Li et al.	Lychee orchard canop	Improved lightweight U-Net (ResNet34 + CBAM + Focal Loss)	Instance segmentation of lychee canopy	[101]
Xue et al.	Citrus canopy	RGB-D images with improved DeepLabv3+ segmentation	Citrus canopy segmentation	[102]
Hu et al.	Apple fruits	RGB-D-based improved YOLOX detection model	Apple fruit detection and localization	[103]
Xu et al.	Peach canopy	RGB-D-based canopy feature encoding	Density map prediction and precision spraying	[104]

**Table 8:** Multispectral-based canopy perception techniques.

Researcher	Research Object	Research Method	Research Objective	Ref.
Kriston-Vizi et al.	Peach tree canopy	Multispectral evaluation	Canopy water stress analysis	[106]
Chandel et al.	Apple tree canopy	Multispectral detection	Apple powdery mildew detection and mapping	[107]
Van Beek et al.	Pear tree canopy	WorldView-2-based multispectral detection	Stem water potential estimation	[108]
Sun et al.	Apple tree canopy	UAV-based multispectral detection	Evaluation of leaf nitrogen status	[109]
Yu et al.	Apple tree canopy	UAV-based multispectral detection	Leaf area index (LAI) measurement	[110]
Tu et al.	Pear tree canopy	Multispectral UAS detection	Canopy structural and physiological condition assessment	[111]

**Table 9:** Variable-rate spraying technologies based on canopy perception.

Researcher	Research Object	Research Method	Research Objective	Ref.
Liu et al.	Orchard tree canopy	Canopy volume perception model	Adjust pesticide dosage based on Pulse-Width Modulation (PWM)	[117]
Xue et al.	Citrus canopy	Canopy volume detection using Kinect senso	Adjust spray flow using PWM based on canopy volume	[118]
Chen et al.	Citrus and litchi canopy	UAV LiDAR + IPTD filtering + region-growing segmentation	Generate prescription maps to guide volume-based spraying	[119]
Jiang et al.	Orchard tree canopy	FAVD-based canopy density estimation	Construct FAVD–spray volume control model with PWM for variable-rate spraying	[120]
Fessler et al.	Apple tree canopy	Laser scanning-based canopy volume and density acquisition	Canopy-driven control + nozzle PWM regulation + fuzzy PID controller for variable-rate pesticide adjustment	[121]
Salas et al.	Orchard tree canopy	Semantic segmentation of canopy regions	Variable spraying based on image semantic segmentation + PWM-controlled variable nozzles + ternary boom design	[122]

**Table 10:** Real-time feedback and closed-loop coordinated control techniques.

Researcher	Research Object	Research Method	Research Objective	Ref.
Jiang et al.	Fruit tree trunk	LiDAR-based navigation; DBSCAN, K-means, and RANSAC algorithms	Autonomous navigation of orchard spraying robot via LiDAR; optimized path planning and precise spraying	[127]
Luo et al.	Kiwifruit canopy	Vision-based canopy detection; ESO fuzzy adaptive control algorithm	Tree canopy feature recognition using machine vision for optimized spray volume and precise control	[128]
Liu et al.	Fruit tree canopy	Single 3D LiDAR sensing; RANSAC algorithm; ROI extraction	Perception of canopy structure to reduce pesticide usage and optimize spray path	[129]
Zhang et al.	Pear tree canopy	Real-time disease spot detection using YOLOv5m CNN; PWM-based nozzle control	Develop a real-time variable-rate spray system based on disease spot level; reduce pesticide use while ensuring application quality	[130]

**Table 11:** Comparison of cost and performance advantages of different canopy perception technologies.

Technology Type	Low-Cost Solutions	High-Cost Solutions	Key Factors Influencing Price Gap	Performance Advantages
Monocular Vision	$70–420	$1120–4200	Resolution (60 fps < 200 fps); low-light performance (20 dB < 50 dB)	Low cost, simple structure; suitable for image-based recognition and contour extraction
Stereo Vision	$350–1120	$2800–7000	Depth estimation accuracy (5% > 1%); effective range (3 m < 20 m)	Enables markerless depth estimation; ideal for mid-range 3D reconstruction
RGB-D Sensors	$252–840	$2100–5600	Point cloud density (50K points < 1M points)	Simultaneous acquisition of color and depth data; facilitates canopy modeling and object identification
LiDAR	$210–1680	$7000–42,000	Angular resolution (1° > 0.1°); penetration rate (30% < 90%)	High point cloud accuracy and strong penetration; suitable for complex canopy perception
Multispectral Sensors	$1120–4900	$8400–35,000	Number of spectral bands (5–12); accuracy (±8% to ±1%)	Capable of detecting plant diseases, pests, and nutrient status; supports intelligent variable-rate spraying and zonal analysis

## Data Availability

No new data were created or analyzed in this study. Data sharing is not applicable to this article.
